# La salud colectiva en el contexto de la bioética global

**DOI:** 10.18294/sc.2023.4737

**Published:** 2023-11-27

**Authors:** Volnei Garrafa, Gabriela Irrazábal, Camilo Hernán Manchola Castillo

**Affiliations:** 1 Profesor Emérito. Miembro, Comité Internacional de Bioética de la Unesco (2010-2017). Docente-fundador, Programa de Posgrado en Bioética, Universidade de Brasília. Brasilia, Brasil. garrafavolnei@gmail.com Universidade de Brasília Universidade de Brasília Brasilia Brazil garrafavolnei@gmail.com; 2 Investigadora adjunta, Consejo Nacional de Investigaciones Científicas y Técnicas, con sede en el Centro de Estudios e Investigaciones Laborales (CEIL), Ciudad Autónoma de Buenos Aires, Argentina. gabrielairrazabal@gmail.com Consejo Nacional de Investigaciones Científicas y Técnicas Consejo Nacional de Investigaciones Científicas y Técnicas Centro de Estudios e Investigaciones Laborales (CEIL) Ciudad Autónoma de Buenos Aires Argentina gabrielairrazabal@gmail.com; 3 Doctor en Bioética. Consultor en bioética y ética en investigación con seres humanos. Profesor, investigador, Universidade de Brasília, Brasilia, Brasil. camilomanchola@gmail.com Universidade de Brasília Universidade de Brasília Brasilia Brazil camilomanchola@gmail.com

Los fundamentos epistemológicos de la bioética cambiaron sustancialmente a partir del año 2005. La aprobación de la Declaración Universal sobre Bioética y Derechos Humanos por la 33a Conferencia General de la Unesco, celebrada en octubre de ese año en París^1^, en la práctica concreta, significó una constancia pública de su reconocimiento y emancipación internacional.

La Unesco dedicó más de dos años de discusiones hasta llegar a un documento final satisfactorio. La homologación de la Declaración ocurrió por aclamación, lo que significa que fue respaldada unánimemente por los 191 países miembros oficiales de la organización en aquel momento. El camino de su construcción, sin embargo, fue largo y tortuoso, entre avances y retrocesos, pasando por diversas versiones preliminares elaboradas por su Comité Internacional de Bioética, un organismo formado por 36 investigadores del área con representación equilibrada entre los cinco continentes.

Si bien el bios de la bioética tiene el significado original de “vida”, hasta octubre de 2004 la dirección dada al documento redujo el tema al campo estrictamente biomédico-biotecnológico, área de interés directo para los países centrales del sistema-mundo que, durante las discusiones, intentaron evitar de diferentes maneras la ampliación de su agenda, de acuerdo con los intereses de los grandes laboratorios internacionales de medicamentos, directamente interesados ​​en el control ético de las investigaciones clínicas. Sin embargo, desde el inicio del proceso de construcción de la Declaración, un grupo orgánico de representantes de países latinoamericanos presionó de forma permanente al Comité Internacional de Bioética para lograr un texto más amplio, afirmativo e inclusivo, expresando su desacuerdo con el rumbo que estaba siendo dado al documento^2^.

En este contexto, a principios de noviembre de 2004, a solicitud de la Secretaría de Derechos Humanos de la República Argentina, con el apoyo de la Red Latinoamericana y del Caribe de Bioética de la Unesco (Redbioética), se llevó a cabo una reunión celebrada en Buenos Aires para discutir formalmente el tema, en la que participaron la entonces presidenta del Comité Internacional de Bioética, la investigadora canadiense Michèle Jean, y algunos de sus miembros, así como expertos de 11 países latinoamericanos, muchos de ellos defendiendo posiciones previamente analizadas por sus países de origen. Del citado encuentro se produjeron dos documentos, ambos contundentes, que abogaban por cambios profundos en el contenido de la Declaración: el primero de ellos, más formal, de perfil técnico, discutía punto por punto el borrador hasta entonces propuesto por el Comité Internacional de Bioética; y el segundo, con el título “Carta de Buenos Aires”^2^^,^^3^, de fuerte base política, criticaba duramente los intereses sectoriales antes mencionados, que fue inmediatamente traducida al inglés y compartida a nivel mundial. A la par de la reunión de Buenos Aires se realizaron con el mismo propósito otras reuniones similares en diferentes países, acción que contribuyó a un cambio del panorama observado hasta entonces con relación al contenido del documento.

La reunión ordinaria del Comité Internacional de Bioética celebrada en París, en enero de 2005, cuando debía definirse el rumbo que la Declaración seguiría a partir de entonces, contó además con la participación oficial de las representaciones diplomáticas de los 36 países que integraban en ese momento el Comité Internacional Intergubernamental de Bioética de la Unesco (CIGB). De la citada reunión, debido a las fuertes posiciones aportadas por los “países periféricos” -denominación geopolítica acuñada por la bioética de intervención^4^- surgió un borrador con bases muy distintas a las anteriores, que pasó a ser el referente para subsidiar las dos reuniones a realizarse en París, en los meses de abril y junio, que fueron decisivas de los llamados “expertos gubernamentales de nivel II” que representarían formalmente a los países componentes de la organización.

El contenido final de la Declaración, presentado para su aprobación en la Conferencia General de la UNESCO el 19 de octubre de ese año, incorporó definitivamente a la agenda bioética del siglo XXI, no solo los temas biomédicos y biotecnológicos (“situaciones emergentes”) ya tratados inicialmente por la práctica bioética, hasta entonces desarrollada en la mayoría de los países, sino también las cuestiones sanitarias, sociales y ambientales (“situaciones persistentes”, de acuerdo con el microsistema lingüístico propuesto por la bioética de intervención), de gran interés para las naciones periféricas del sistema-mundo. En otras palabras: la lucha de los países latinoamericanos, acompañada de la casi totalidad de las naciones africanas, los países del sur de Asia y la mayoría de los países árabes, entre otros, contribuyó decisivamente al cambio definitivo, la ampliación y la politización de la agenda bioética internacional.

Las personas menos familiarizadas con la bioética podrían preguntarse cuáles fueron las razones para la construcción de nuevos referentes conceptuales de apoyo a la disciplina si ya habían sido definidos desde la proposición del neologismo bioética, en 1970, por Van Rensselaer Potter^5^ y la utilización de los denominados “cuatro principios de Georgetown” como su herramienta metodológica hegemónica. Inmediatamente después de la presentación del libro *Bioethics: bridge to the future*^6^*,* que en la práctica dio origen a la bioética, otros investigadores y organizaciones modificaron la idea original de Potter, con una fundamentación esencialmente biomédica aplicada sobre todo a las situaciones de conflictos en la relación de los profesionales de salud con sus pacientes, y de los investigadores y laboratorios farmacéuticos con las personas-sujetos de las investigaciones clínicas. En cambio, Potter había imaginado la bioética con una visión de “puente”, como una ética relacionada con los fenómenos de la vida humana en su sentido más amplio, que incorporaba no solo cuestiones biomédicas y sociales, sino especialmente temas ambientales vinculados a la sostenibilidad del planeta. Un poco más tarde, en 1988, para renovar y reforzar sus ideas, el autor empezó a denominarla “bioética global”^7^.

En 1978 apareció la *Encyclopedia of bioethics*^8^, editada en EEUU por Warren Reich, que redujo la bioética original potteriana a temas esencialmente biomédicos que, como ya se mencionó, no constituían las cuestiones centrales ni mucho menos las únicas propuestos por Potter. Y fue con este nuevo enfoque, ortodoxo y profundamente ligado a las raíces culturales anglosajonas, que la bioética se dio a conocer internacionalmente en la década de 1980, consolidándose definitivamente en la década siguiente.

La epistemología de esta bioética, con base médica y clínica, tomó como referencia los principios de “respeto a la autonomía” de las personas, “beneficencia” (hacer el bien) y “justicia”, emanados del conocido “Informe Belmont”^9^, documento solicitado por el gobierno estadounidense a un comité de expertos del país con el objetivo de contener las transgresiones éticas que venían ocurriendo reiteradamente en ese país desde el periodo posguerra con relación a las investigaciones desarrolladas en seres humanos. Poco después, en 1979, Tom Beauchamp y James Childress publicaron la primera edición de la obra *Principles of Biomedical Ethics*^10^, considerada un referente de la denominada “bioética principialista” anglosajona de origen estadunidense, que incorpora un cuarto principio a los tres ya mencionados: la “no-maleficencia” (del juramento hipocrático *primum non nocere*, ante todo, no hacer daño). Estos cuatro principios, presumiblemente universales, se popularizaron en todo el mundo, llegando a confundirse e incluso entenderse como la propia bioética.

En la década de 1990, sin embargo, empezaron a surgir críticas a esta corriente de pensamiento bioético (que acabó conociéndose como principialismo) y la universalidad de sus principios, con base ​​en dos interpretaciones diferentes. La primera crítica, proveniente del norte (EEUU y Europa), analizó conceptualmente sus aspectos filosóficos y su validez como teoría^11^^,^^12^^,^^13^; mientras que la segunda, de origen latinoamericana, procuro demostrar que la aplicación directa de los cuatro principios a las diferencias concretas existentes entre los países centrales y periféricos, contribuía asépticamente al mantenimiento de las flagrantes asimetrías encontradas entre ricos y pobres del planeta^14^^,^^15^.

A partir del Cuarto Congreso Mundial promovido por la International Association of Bioethics (IAB) celebrado en Tokio, Japón, en 1998, la bioética (re)comenzó a tomar otros caminos, a partir del tema oficial del evento que fue “Bioética Global”. Con una fuerte influencia del investigador escocés Alastair Campbell, entonces presidente de la IAB, parte de los seguidores de la bioética retomaron los caminos originales trazados por Potter quien, ya muy mayor y enfermo, envió una videoconferencia presentada en la apertura del Congreso, donde reafirmó sus convicciones originales.

Reforzando lo ya dicho, hasta 1998, la epistemología de la bioética se restringía a caminos que apuntaban centralmente a temas biomédico-biotecnológicos en detrimento de los sociosanitarios y ambientales, y a problemas/conflictos preferentemente individuales en relación con los colectivos. La maximización y el sobredimensionamiento del principio de autonomía convirtió al principio de justicia en un mero elemento de apoyo de la teoría principialista; una especie de apéndice, aunque indispensable, de menor importancia. Lo individual asfixió al colectivo, el “yo” de la autonomía terminó dejando al “nosotros” de la justicia en una posición secundaria. 

En otras palabras, la teoría principialista se mostraba incapaz o impotente para desvelar, comprender, proponer soluciones e intervenir en los agudos problemas socioeconómicos y de salud colectiva que persisten en la mayoría de los países periféricos del sur del mundo^4^. En cierto modo, el principio de justicia significó para el principialismo, proporcionalmente, lo que la promoción de la salud representó en los años 1960 y 1970 para la llamada “medicina social”: algo en cierta medida menos importante y solamente de apoyo, donde caben todos los demás problemas no contenidos en sus otras categorías teóricas consideradas entonces como principales (protección específica, diagnóstico precoz, tratamiento y rehabilitación).

El Sexto Congreso Mundial de Bioética, realizado en Brasilia en 2002 con la participación de 1.400 investigadores de 62 países, fortaleció la voz de quienes no estaban de acuerdo con el desequilibrio observado en la balanza, a partir de la definición del tema del evento, que fue “Bioética, Poder e (In)justicia”. Las fuertes discusiones registradas en el evento sacaron a la luz la necesidad de que la especialidad pasase a incorporar a su campo de reflexión y acción aplicada, temas sociopolíticos que persistían insistentemente en aquel momento histórico, principalmente las agudas discrepancias sociales y económicas existentes entre ricos y pobres, entre naciones geopolíticamente ubicadas al norte o al sur del planeta. Según Daniel Wickler, ex presidente de la IAB y primer consultor en bioética de la Organización Mundial de la Salud en la década de 1990, “...la combinación entre bioética y política es nueva y saludable para el área… Fue una hazaña histórica que significó un gran impulso a la bioética en América Latina y en el mundo”^2^^,^^16^.

A finales del siglo XX y principios del siglo XXI, por tanto, la bioética amplió su campo de estudio y acción, incorporando a su contexto cuestiones directamente relacionadas con la calidad de vida humana, temas que hasta entonces solo tocaban tangencialmente su agenda, como: derechos humanos y ciudadanía; derecho al acceso a la salud; priorización en la asignación de recursos sanitarios escasos; la preservación de la biosfera y la biodiversidad; la finitud de los recursos naturales planetarios; el equilibrio del ecosistema; los alimentos transgénicos; el racismo y otras formas de discriminación; etc.

Con las transformaciones y el nuevo ritmo que comenzó a vivirse en el contexto internacional de la bioética, el ámbito de la ética aplicada dejó de ser considerado algo de carácter supraestructural, meramente individual o específico para pasar a incluir la participación directa de la sociedad civil organizada en las discusiones con vistas al bienestar futuro de las personas y comunidades. La cuestión ética, por tanto, adquirió una identidad pública; dejó de ser considerada solo una cuestión de conciencia a ser resuelta en el ámbito privado o particular, de foro individual y exclusivamente íntimo. Hoy en día, adquiere una importancia creciente en lo que se refiere especialmente al análisis de las responsabilidades públicas y sanitarias y a la interpretación histórico-social más precisa de las condiciones epidemiológicas, además de ser esencial para determinar las formas de intervención a programar, en el respeto al equilibrio ambiental, en la formación adecuada del personal sanitario, en la responsabilidad de los Estados hacia los ciudadanos, especialmente los más frágiles y necesitados.

Y fue en este contexto -y como consecuencia- que surgieron organizaciones regionales o incluso internacionales como la Redbioética, la International Association for Education in Ethics (IAEE) y otras, con el objetivo de producir nuevos conocimientos teórico-prácticos relacionados con la bioética global, más comprometidos con las cuestiones concretas que afectan a las poblaciones mayoritarias de los países pobres y en desarrollo del mundo. Salvo honrosas excepciones, lamentablemente las “naciones periféricas” están acostumbradas, en los más diversos campos, a importar horizontal y acríticamente conocimientos y tecnologías de las “naciones centrales” industrializadas, a menudo con consecuencias desastrosas para los pueblos receptores de las novedades. Recientemente, hasta “paquetes éticos” comenzaron a importarse verticalmente, de arriba hacia abajo, sin ningún filtro crítico, como en el caso del principialismo.

En contra de la masificación descontextualizada de la información científica que coloniza a los pueblos periféricos del mundo, estas nuevas organizaciones comenzaron a desarrollar acciones críticas a través de la promoción de diferentes actividades, eventos y publicaciones, privilegiando el respeto a las raíces culturales autóctonas, de manera orgánica y socialmente comprometida que incluya una sana apertura para diálogos académicos bilaterales; aprovechando de manera sana el conocimiento y las experiencias que vienen de afuera, pero solamente después de sometidos a un riguroso filtro.

Por otro lado, y relacionado con las “situaciones emergentes” referidas por la bioética de intervención (situaciones límites del desarrollo científico-tecnológico), los actuales Objetivos de Desarrollo Sostenible (ODS) de la agenda 2030 de Naciones Unidas, que establecen un marco para “…garantizar una vida sana y promover el bienestar para todos en todas las edades”^17^, reconocen el potencial del desarrollo de las tecnologías de la información y comunicación (TIC) para la salud y el bienestar social, la necesidad de reducción de la brecha digital y la urgencia en la promoción de sociedades del conocimiento. En la última década, la industria y el mercado de la salud digital han experimentado un significativo crecimiento en tamaño y ganancias económicas. Esta expansión puede observarse en el desarrollo de ecosistemas biotecnológicos de salud digital en diferentes países, alianzas entre empresas, gobiernos y universidades para el desarrollo de tecnologías de salud. 

La salud digital, que incluye la telemedicina, los dispositivos portátiles, la infraestructura de datos de salud, las aplicaciones móviles de ¨bienestar y salud¨ (médicas) y la inteligencia artificial, se presenta como el potencial de mejora de la atención médica a través de las TIC, alineándose con la estrategia de la OMS y los Objetivos de Desarrollo Sostenible. Sin embargo, persisten preocupaciones sobre la desigualdad y la exclusión ya que la brecha digital delimita un “superdeterminante social de la salud”. El debate (bio)ético plantea preguntas sobre si este escenario representa injusticias globales hacia el establecimiento de un futuro inclusivo y perpetua las desigualdades en la salud colectiva. Las personas y países, entonces, se encuentran en la encrucijada por acceder a la atención médica y lograr un bienestar general en el marco de procesos desiguales de transición digital de los sistemas de salud y, especialmente, de serias amenazas al respeto por su vulnerabilidad y privacidad.

La afirmación de todo esto puede comprobarse por el contenido de los 15 principios incluidos en los 28 artículos de la Declaración Universal sobre Bioética y Derechos Humanos^1^, entre los que se incluyen un conjunto de temas directamente relacionados con la salud pública y que son objeto de esta convocatoria, como los siguientes: Respeto de la vulnerabilidad humana y la integridad personal (Art. 8); Igualdad, justicia y equidad (Art. 10); No discriminación y no estigmatización (Art. 11); Respeto de la diversidad cultural y del pluralismo (Art. 12); Solidaridad y cooperación (Art. 13); Responsabilidad social y salud (Art. 14); Aprovechamiento compartido de los beneficios (Art. 15); Protección de las generaciones futuras (Art. 16); y Protección del medio ambiente, la biosfera y la biodiversidad (Art. 17).

En el contexto específico de este editorial, vale la pena registrar el contenido completo del Artículo 14 de la Declaración Universal sobre Bioética y Derechos Humanos^1^, que trata de la “Responsabilidad social y salud”:

1) La promoción de la salud y el desarrollo social para sus pueblos es un cometido esencial de los gobiernos, que comparten todos los sectores de la sociedad.
2) Tomando en cuenta que el goce del grado máximo de salud que se pueda lograr es uno de los derechos fundamentales de todo ser humano sin distinción de raza, religión, ideología política o condición económica o social, los progresos de la ciencia y la tecnología deberían fomentar:
a) el acceso a una atención médica de calidad y a medicamentos esenciales, especialmente para la salud de las mujeres y los niños, ya que la salud es esencial para la vida misma y debe considerarse un bien social y humano;
b) el acceso a una alimentación y un agua adecuados;
c) la mejora de las condiciones de vida y del medio ambiente;
d) la supresión de la marginación y exclusión de personas por cualquier motivo; y
e) la reducción de la pobreza y el analfabetismo.

En resumen, la Declaración Universal sobre Bioética y Derechos Humanos de la Unesco^1^, junto con otras iniciativas más puntuales desarrolladas en las últimas décadas, cambiaron sustancialmente la agenda de la disciplina en el siglo XXI, con la incorporación de temas persistentes y emergentes de interés directo para las naciones periféricas del planeta, principalmente las del hemisferio sur del mundo, a través de la construcción de nuevos referentes epistemológicos más cercanos a la realidad donde la bioética ha sido llamada a actuar e intervenir cada día con más frecuencia. 
